# Relationship Between Vitamin D Insufficiency and Anemia in Older Adults: An Approach Considering Clinical Aspects and Food Insecurity

**DOI:** 10.3390/nu16213669

**Published:** 2024-10-28

**Authors:** Maria Cecília Cougo Mesquita, Rafaela Martins de Castro, Talissa Vicente Mendes, Mariana Araújo Vieira do Carmo, Eliza de Souza Sampaio, Ligiana Pires Corona, Daniela Braga Lima, António Raposo, Ibrahim Alasqah, Nada Alqarawi, Najla A. Albaridi, Zayed D. Alsharari, Tábatta Renata Pereira de Brito

**Affiliations:** 1 Faculty of Nutrition, Federal University of Alfenas, Alfenas 37130-001, Brazil; maria.mesquita@sou.unifal-mg.edu.br (M.C.C.M.); rafaela.castro@sou.unifal-mg.edu.br (R.M.d.C.); talissa.mendes@sou.unifal-mg.edu.br (T.V.M.); marianavieira06@hotmail.com (M.A.V.d.C.); eliza.sampaio@sou.unifal-mg.edu.br (E.d.S.S.); daniela.lima@unifal-mg.edu.br (D.B.L.); 2 Applied Sciences School, University of Campinas, Campinas 13083-970, Brazil; ligiana.corona@fca.unicamp.br; 3 CBIOS (Research Center for Biosciences and Health Technologies), Universidade Lusófona de Humanidades e Tecnologias, Campo Grande 376, 1749-024 Lisboa, Portugal; 4 Department of Psychiatric and Mental Health, and Community Health, College of Nursing, Qassim University, Buraydah 51452, Saudi Arabia; i.alasqah@qu.edu.sa (I.A.); n.alqarawi@qu.edu.sa (N.A.); 5 Department of Health Science, College of Health and Rehabilitation, Princess Nourah bint Abdulrahman University, P.O. Box 84428, Riyadh 11671, Saudi Arabia; naalbaridi@pnu.edu.sa; 6 Department of Clinical Nutrition, Faculty of Applied Medical Sciences, University of Tabuk, P.O. Box 741, Tabuk 71491, Saudi Arabia; zalsharari@ut.edu.sa

**Keywords:** anemia, elderly, food insecurity, hemoglobin, nutrition, nutritional deficiency, vitamin D

## Abstract

Background/Objectives: Studies have shown a high prevalence of anemia and vitamin D insufficiency in older adults, and the literature suggests a relationship between these two conditions, as vitamin D insufficiency may impair erythrocyte synthesis. Food insecurity refers to the lack of regular access to sufficient and nutritious food, which can directly affect health by worsening conditions such as anemia and vitamin D insufficiency. This study evaluated the association between vitamin D insufficiency and anemia in older adults. Methods: We conducted a cross-sectional study with 430 individuals aged 60 and older, using personal interviews and blood tests for data collection. Anemia was identified with serum hemoglobin levels of <12 g/dL for women and <13 g/dL for men, while vitamin D insufficiency was defined as serum levels <30 ng/mL. We used multiple logistic regression to analyze associations through Stata version 17.0 software. Results: The prevalence of anemia was identified in 14.7% of the sample, and vitamin D insufficiency was observed in 63.5%. We found an association between vitamin D insufficiency and anemia (OR = 2.4; 95% CI = 1.2–4.7). In the final model, factors such as male sex (OR = 2.7; 95% CI = 1.5–4.9) and polypharmacy use (OR = 2.0; 95% CI = 1.0–3.9) were also associated, regardless of age group, food insecurity, and multimorbidity. Conclusions: Vitamin D insufficiency increased the likelihood of anemia among the older adults evaluated, suggesting that prevention and treatment strategies for anemia should consider vitamin D serum levels.

## 1. Introduction

Population aging is a process characterized by declining birth and mortality rates and a consequent increase in life expectancy [1]. As life expectancy rises, the number of older adults living with noncommunicable chronic diseases also grows, a trend highlighted by demographic shifts and changes in the population’s epidemiological profile [2,3].

Research has shown that low serum levels of 25-hydroxyvitamin D [25(OH)D] may be linked to an increased risk of developing various chronic conditions, such as diabetes, hypertension, hyperlipidemia, cardiovascular disease, cancer, and autoimmune diseases—conditions that represent major global public health challenges [4,5,6,7,8,9]. Studies reveal that vitamin D inhibits the growth of cancer cells by inducing apoptosis, as discussed by Muñoz and Grant (2022) [5]. Daily vitamin D supplementation to maintain a serum 25(OH)D level ≥ 100 nmol/L is a promising approach to reduce the risk of diabetes in adults with prediabetes [10]. The findings of systematic reviews and meta-analyses underscore the need for personalized vitamin D intervention strategies that comprehensively account for individual patient characteristics (such as ethnocultural background, age, body mass index, and circulating 25[OH]D level), intervention dosage, and intervention duration to optimize cardiometabolic health outcomes [11]. The joint recommendation of the Brazilian Society of Clinical Pathology/Laboratory Medicine (SBPC/ML) and the Brazilian Society of Endocrinology and Metabology (SBEM) suggests that plasma levels of 25(OH)D should be greater than 20 ng/mL for the general healthy population and between 30 and 60 ng/mL for at-risk groups, such as older adults, pregnant women, pre-bariatric patients, and individuals with osteomalacia, rickets, osteoporosis, secondary hyperparathyroidism, inflammatory diseases, autoimmune diseases, and chronic kidney disease. Rolizola et al. observed a prevalence of vitamin D insufficiency in 64.5% of 533 older participants with a mean age of 69.6 years [7].

Another condition that warrants attention in older adults is anemia, defined as a reduction in the number of red blood cells or in their capacity to transport oxygen via hemoglobin to meet physiological needs. Anemia is frequently diagnosed in older individuals [8,9], and its etiology at an advanced age is complex, ranging from bone marrow failure syndromes, chronic kidney disease, and nutritional deficiencies to inflammatory processes, including inflammation in immunosenescence [9].

The anatomical, physical, and physiological changes resulting from aging directly affect this health population’s health and nutrition. Changes in taste, as well as in hormonal and digestive secretions of the gastrointestinal tract, lead to alterations in eating habits and nutritional status [12,13,14], which may contribute to anemia. In older adults, anemia can be classified into three categories according to its cause: (1) nutritional deficiency (iron deficiency is the leading cause of anemia due to the essential role of iron in oxygen transport and its low availability in the diet for a large portion of the global population); (2) secondary to a noncommunicable chronic condition; or (3) unexplained, which increases the risk of morbidity and mortality [15]. Anemia represents a public health issue that is expected to rise among older adults in the coming years due to demographic shifts. Unfortunately, this condition is often neglected, especially in older individuals with multimorbidity, and in many cases, it remains undiagnosed and untreated [8].

A study based on secondary laboratory data from the 2013 National Health Survey indicated that anemia affected approximately 9.9% of Brazil’s adult and older population [16]. The disease is marked by a decrease in hemoglobin concentration in the blood, diagnosed when levels fall below 12.0 g/dL for women and 13.0 g/dL for men [17]. In older adults, anemia increases hospitalizations, cognitive decline, falls, and fracture risks [18,19].

**Figure 1:**
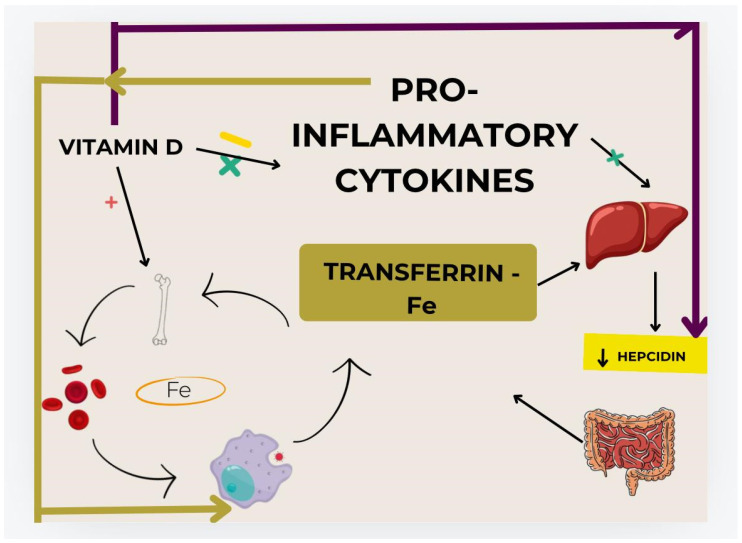
Proposed role of vitamin D in enhancing iron recycling (adapted from Smith and Tangpricha [25]).

Evidence in the literature show that individual exposure to the sun, accumulation of fat mass, skin type, physical activity, dietary intake of vitamin D, and genetic markers are important factors associated with low serum levels of 25(OH)D [20,21,22] and suggests that vitamin D insufficiency may contribute to increased pro-inflammatory cytokine levels and impaired red blood cell production. Additionally, hepcidin, a liver hormone involved in iron homeostasis, plays a role in this process, as vitamin D can enhance iron absorption by regulating the iron–hepcidin–ferroportin axis (Figure 1). Therefore, vitamin D deficiency may promote the development of anemia [23,24].

As illustrated in Figure 1, iron recycling, under non-pathologic conditions, involves transferrin-bound iron in circulation traveling to the bone marrow to support erythropoiesis. Upon senescence, red blood cells (RBCs) are engulfed by macrophages and iron is recycled back into circulation to support further erythropoiesis. Dietary iron may also enter the circulating pool from absorption in the duodenum based on the body’s needs. Elevations in pro-inflammatory cytokines suppress erythropoiesis in the bone marrow and shorten RBC lifespan due to increased macrophage activation and erythrophagocytosis. Pro-inflammatory cytokines IL-6 and IL-1β also stimulate the liver to upregulate the expression of hepcidin antimicrobial peptide (HAMP). Hepcidin inhibits iron egress from cells of the reticuloendothelial system, including enterocytes and macrophages, by binding to and inducing the degradation of the cellular iron exporter, ferroportin, resulting in decreased iron absorption from the small intestine and increased iron sequestration within the macrophage. Vitamin D has been shown to promote erythropoiesis and iron recycling by increasing erythroid progenitor proliferation, decreasing pro-inflammatory cytokines, and suppressing hepcidin expression. Vitamin D may directly reduce circulating hepcidin concentrations independent of changes in inflammatory cytokines. Decreases in pro-inflammatory cytokines and hepcidin may increase iron bioavailability for erythropoiesis and hemoglobin synthesis by preventing iron sequestration in macrophages and removing impairments to iron absorption, thus restoring iron recycling.

Clinical conditions such as multimorbidity, characterized by the presence of multiple chronic diseases in older individuals, may intensify the association between vitamin D insufficiency and anemia. Chronic conditions like diabetes, hypertension, cardiovascular disease, and osteoporosis are often linked to inflammatory states and elevated pro-inflammatory cytokines, which may interfere with red blood cell production and contribute to the development of anemia. Furthermore, multimorbidity may limit sun exposure and reduce the absorption and production of vitamin D, leading to a deficiency that hinders iron metabolism and hemoglobin production [26]. Consequently, older adults with multiple chronic diseases are more likely to experience both vitamin D insufficiency and anemia.

Food insecurity is another important factor that may exacerbate the relationship between vitamin D insufficiency and anemia in older individuals. Difficulty accessing nutritious foods rich in vitamin D and iron contributes to insufficient intake of these essential nutrients, worsening nutritional deficiencies. Studies have shown that food insecurity is associated with a higher risk of anemia due to inadequate consumption of iron-rich foods and other nutrients necessary for hemoglobin production [27]. Thus, the combination of multimorbidity and food insecurity may amplify the association between vitamin D insufficiency and anemia in older adults, as both conditions negatively affect this population’s nutritional status and overall health.

In this context, considering the importance of further research to elucidate the relationship between vitamin D insufficiency and anemia, alongside the scarcity of studies involving older populations, the present study aimed to evaluate the association between vitamin D insufficiency and anemia in older adults.

## 2. Materials and Methods

### 2.1. Study Design, Setting, and Population

This study is part of a research project titled “Association between Low Levels of Social Support and Telomere Length in Older Adults” [28]. It represents a quantitative investigation employing an analytical cross-sectional design. The design and reporting adhered to the principles set forth by the STROBE (Strengthening the Reporting of Observational Studies in Epidemiology) initiative.

We conducted the study in the city of Alfenas, in the southern region of Minas Gerais, Brazil. According to IBGE estimates (2019), Alfenas had a population of 79,996 in 2019 [29]. At the time of sampling, the most recent age-related population projection available, provided by RIPSA (Interagency Health Information Network), dated back to 2015 and estimated that there were approximately 10,797 older adults within a total population of 78,713 inhabitants (Brazil, 2020) [30].

**Figure 2:**
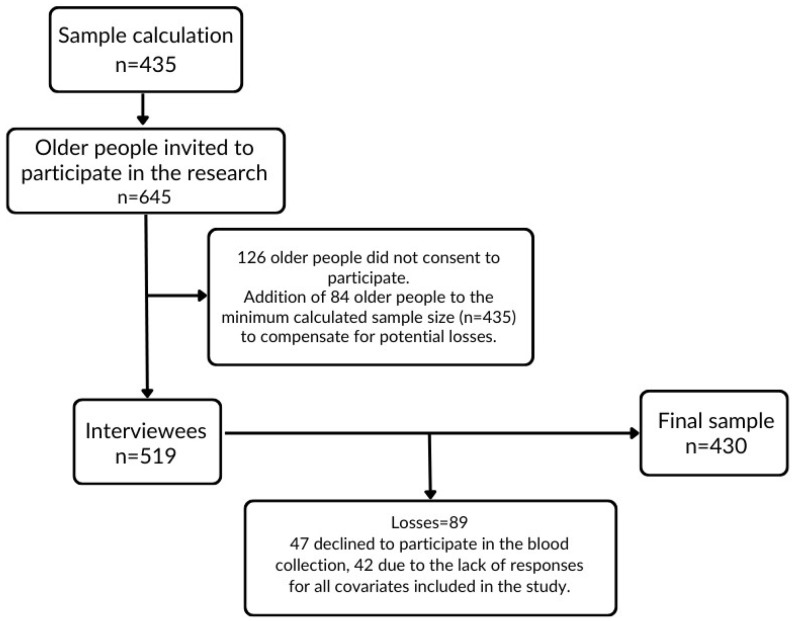
Sample definition.

The study population consisted of individuals aged 60 and older who resided in the urban area of Alfenas in 2019. The sample size calculation was based on an estimated proportion of 50%, a 95% confidence interval, a design effect of 1.17, and a population of 10,797 older adults. The final sample included 430 individuals (Figure 2).

We selected older adults to participate in the study based on strategically chosen households to ensure the inclusion of individuals from all areas of the municipality. This selection process was inspired by the sampling method used in the Health, Well-being, and Aging Study (SABE), a population-based survey conducted in the city of São Paulo [31]. Interviewers were assigned to various city regions, considering the proximity of their homes. When interviewers identified a household with a resident aged 60 or older, they sought nearby households or, at most, others within the same neighborhood.

To be included in the study, participants had to meet the following inclusion criteria: be at least 60 years old and able to respond to the questionnaire. The interviewer assessed this ability through the participant’s capacity to answer personal information such as name, date of birth, address, and phone number during the research presentation and invitation to participate. If the potential participant could not answer at least one of these questions, they were deemed ineligible for the study. The exclusion criterion was a permanent or temporary disability that affected mobility unless the individual could use a mobility aid. This criterion was applied due to the physical tests conducted in the larger study, which required a minimum level of mobility.

Data collection occurred in two distinct phases between July and December 2019. In the first phase, we conducted interviews and physical assessments. In the second phase, blood samples were collected after a 12 h fast. The interviews were conducted at the participants’ homes, and blood samples were collected at the Central Laboratory of Clinical Analyses (LACEN) at the Federal University of Alfenas (UNIFAL-MG) or the participants’ homes, depending on their ability to travel to LACEN.

This study was conducted following the Declaration of Helsinki, and informed consent was obtained from all participants, ensuring their privacy rights were respected. The study was submitted to the Ethics Committee on Human Research and approved under opinion number 2.668.936/2018, CAAE: 85218518.0.0000.5142.

### 2.2. Measures

The dependent variable in this study was anemia, identified through hemoglobin measurements obtained via a complete blood count. We followed the World Health Organization (WHO) guidelines, defining anemia as hemoglobin levels below 12.0 g/dL for women and below 13.0 g/dL for men [32]. The key independent variable was vitamin D insufficiency, determined by measuring serum 25(OH)D levels through chemiluminescence in blood samples. Vitamin D insufficiency was defined as serum 25(OH)D levels below 30 ng/mL [26,33].

Descriptive and adjustment variables included sex (female; male); age group (60–69 years; 70–79 years; 80 years and older); years of education (≤4 years; >4 years); household income (>2 minimum wages; between 1 and 2 minimum wages; <1 minimum wage); marital status (with a partner; without a partner); living arrangement (does not live alone; lives alone); multimorbidity (0 or 1 disease—no; 2 or more diseases—yes); polypharmacy (0 to 4 medications—no; 5 or more medications—yes); and food insecurity screening (secure; insecure) assessed through the short version of the Brazilian Food Insecurity Scale (EBIA). This is a scale adapted and validated by Santos et al. (2014) for screening food insecurity. It consists of five questions addressing concerns about food availability at home and the financial ability to access it. These questions do not consider the presence of minors in the household and are answered using a dichotomous scale (yes/no), referencing the three months prior to the interview. The household was classified as “food insecure” if the individual responded “yes” to at least one question [34].

### 2.3. Statistical Analysis

We performed the statistical analyses using Stata software, version 17.0. Descriptive analyses estimated proportions, and differences between groups were assessed using Pearson’s χ^2^ and Fisher’s exact tests. We employed multiple logistic regression to analyze associations, with the magnitude of associations estimated through crude and adjusted odds ratios (ORs). Variables with a *p*-value < 0.20 in the univariate analysis were included in the final model via stepwise forward selection, and a significance level of 5% was applied to all analyses.

## 3. Results

Among the 430 older adults evaluated, 70.8% were female, and 45.3% were between 60 and 69 years. The prevalence of anemia was 14.7%, while vitamin D insufficiency affected 63.5% of the population. Regarding other variables, 66.1% of the sample had less than four years of education, 52.3% had a partner, 81.5% did not live alone, 44.3% had a household income between one and two minimum wages, 69.8% experienced multimorbidity, 41.6% were using multiple medications, and 13.6% were experiencing food insecurity.

Table 1 shows the differences in proportions of older adults with anemia regarding sex, polypharmacy, and vitamin D insufficiency. Older men, those taking multiple medications, and individuals with insufficient serum 25(OH)D levels had a higher prevalence of anemia compared to women, older adults with adequate plasma 25(OH)D levels, and those not taking five or more medications. 

In the univariate analysis, men (OR = 2.0; 95% CI = 1.16–3.41; *p* = 0.01), individuals taking five or more medications (OR = 2.0; 95% CI = 1.17–3.43; *p* = 0.01), and those with vitamin D insufficiency (OR = 2.0; 95% CI = 1.06–3.65; *p* = 0.03) were more likely to have anemia (Table 2).

After including variables with a *p*-value < 0.20 in the univariate analysis, the final model revealed that male sex (OR = 2.7; 95% CI = 1.5–4.9), polypharmacy (OR = 2.0; 95% CI = 1.1–3.9), and vitamin D insufficiency (OR = 2.4; 95% CI = 1.2–4.7) were factors independently associated with anemia, regardless of age group, food insecurity, and multimorbidity (Table 3).

## 4. Discussion

Vitamin D has long been recognized for its role in regulating calcium, phosphorus, and bone metabolism. However, it has gained attention in recent years for its involvement in a variety of biological functions, including immune response, cell proliferation, and cardiovascular function. Common among older adults, vitamin D insufficiency is a risk factor for the development of several conditions, such as cardiovascular disease, metabolic disorders, and cancer. Our study demonstrated that vitamin D insufficiency increased the likelihood of anemia in the older population, highlighting that both vitamin D deficiency and anemia are significant public health issues.

A cross-sectional study conducted by Perlstein et al. in the United States, which included 5100 individuals over the age of 60, identified a significant association between 25 hydroxyvitamin D deficiency and the presence of anemia, particularly inflammatory anemia, regardless of age, sex, or race/ethnicity. Among the participants, those with vitamin D deficiency had a higher prevalence of anemia (approximately 70%) than those with normal serum levels (12.3% vs. 7.4%) [35].

In another cross-sectional study conducted in the United States, involving a general sample of 1661 patients with chronic kidney disease, with an average age of 70 ± 11 years, 41% of the individuals met the criteria for anemia diagnosis. The study observed a gradual decline in hemoglobin concentrations and an increase in anemia prevalence as vitamin D concentrations decreased across tertiles. These associations remained significant even after multivariate analysis [36].

Research on the association between vitamin D insufficiency and anemia in older adults is scarce. Although there are differences among the studies regarding the descriptive adjustment variables used and the specific characteristics of the population studied, all suggest that vitamin D insufficiency may be a predisposing factor for anemia. The literature points to three possible physiological mechanisms that explain the association between anemia and vitamin D insufficiency: modulation of pro-inflammatory cytokines, regulation of hepcidin levels, and a reduced response to erythropoietin. These studies suggest that such an association is linked to anemia of inflammatory origin [25]. In this context, the literature suggests that vitamin D can modulate cytokine production in the body, potentially reducing the inflammatory environment [24] and the risk of inflammation-related anemia. Excessive pro-inflammatory cytokines can increase hepcidin expression, which in turn can reduce erythropoiesis and the lifespan of red blood cells [25]. Therefore, hepcidin plays a crucial role in the association between vitamin D deficiency and anemia [37].

Hepcidin is a peptide synthesized mainly by hepatocytes; it regulates iron absorption in the intestine and iron release from cells of the reticuloendothelial system, including enterocytes and macrophages, by binding to ferroportin [38]. In pro-inflammatory states, cytokines such as IL-6 and IL-1β stimulate the liver to upregulate hepcidin expression, leading to poor iron absorption and the trapping of stored iron within immune system cells. This results in insufficient iron availability for erythropoiesis and hemoglobin synthesis [25], promoting the development of anemia of inflammatory origin. However, modulating pro-inflammatory cytokines by vitamin D may suppress hepcidin gene expression and lead to proper iron metabolism regulation [37]. Vitamin D receptors have also been identified in various tissues, such as bone marrow. When activated by vitamin D in the bone marrow, these receptors inhibit the production of pro-inflammatory cytokines, such as IL-1 and IL-6, while upregulating the expression of the anti-inflammatory cytokine IL-10, leading to a decrease in inflammatory processes [23].

Inflammatory cytokines further impair the erythropoiesis process by reducing erythropoietin production and interfering with the differentiation and proliferation of erythroid progenitor cells. In this regard, evidence shows that vitamin D is crucial in erythropoiesis, as it enhances the proliferation of erythroid burst-forming units and has a synergistic effect with erythropoietin, resulting in increased proliferation of erythroid progenitor cells [25]. Given that vitamin D helps modulate the synthesis and secretion of pro-inflammatory cytokines, which raise the risk of anemia, adequate serum levels of 25(OH)D may have a protective effect against this hematological condition.

The prevalence of anemia in our sample was 14.7%, which aligns with a study conducted in Campina Grande, Paraná, Brazil, where anemia prevalence was 12.5% among 360 older adults [39]. The prevalence of vitamin D insufficiency observed in our study was 63.5%, similar to that found in research involving older adults attending Primary Health Care services, reaching 64.5% [7].

Regarding the sample profile, the results are consistent with other population-based studies, such as the SABE (Health, Well-being, and Aging Study) and the ELSI-Brazil (Brazilian Longitudinal Study of Aging). In both these studies and our research, the majority of older individuals interviewed were female, lived with companions, had relatively low household income and low education levels, and were taking medication at the time of the interview [29,39]. These characteristics are concerning since they are directly related to reduced quality of life and influenced by gender, socioeconomic level, education, and health conditions [31]. Our study also found that the prevalence of anemia was higher among men (21.4%), which may indicate the physiological reduction in testosterone levels, a hormone that stimulates erythropoiesis [40]. In a study conducted with 4499 individuals aged 40 and over in South Africa, anemia prevalence increased with age only in men, and the decrease in hemoglobin levels with age in men was not explained by comorbidities, inflammation, or limitations in activities of daily living [41].

Although our findings did not show an association between food insecurity and the outcomes studied, it is important to highlight that studies conducted in recent years have revealed that inadequate and monotonous diets among older adults can affect their nutritional status. This dietary pattern is characterized by an excess of foods rich in sugars and poor in micronutrients, as well as reduced consumption of beans and beef, combined with a low intake of fruits and vegetables. Such habits are recognized as contributing factors to negative health outcomes, including anemia and vitamin D deficiency [42].

Due to its high prevalence and health consequences, addressing anemia remains a priority in public health, being politically supported by Brazil’s commitment to reducing this condition at both national and international levels. This finding may have occurred because the analyses did not consider the different levels of food insecurity (mild, moderate and severe) and grouped all individuals into a single category, food insecurity. The literature points out that food insecurity may be related to some negative health outcomes, such as diabetes, chronic pain, and kidney disease, but with other health problems like micronutrient deficiency, this association does not occur [43,44]. Even so, there is still a discussion about the causality of food insecurity in relation to anemia and vitamin D deficiency, as it requires more effort to deepen the relationship, as most studies on anemia are focused on children and women of reproductive age [27,45]. The goal is to ensure mutual accountability in line with global nutrition objectives, such as eradicating hunger and preventing all forms of malnutrition, as outlined in the 2030 Agenda for Sustainable Development. For Brazil to meet these agreed-upon goals, actions in the food and nutrition security field must be intensified, with special attention to vulnerable groups, including older adults [5]. 

## 5. Limitations

This study presented limitations regarding its design, sample size, lack of specific biochemical tests to identify the type of anemia, reliance on self-reported data, and absence of variables to assess sun exposure, the level of skin pigmentation, and vitamin D supplementation. In the same way, Rolizola et al. (2022) presented some self-reported data, information that is subject to under- or overestimation [7]. In their study, Machado et al. (2019) also pointed out that the sample size was a limitation to estimate the prevalence of anemia in some age and skin-color groups [16], while Bezerra et al. (2022) highlighted that the combination of genetic variants was limited for the same reason [21]. Moreover, as for the variables, we used multimorbidity and polypharmacy in the present study precisely because they include all diseases and medications reported by the older adult participants, although, herein, elderly people with some chronic conditions such as intestinal malabsorption, chronic hepatic disease, and renal impairment were not excluded but might be expected to develop vitamin D deficiency and/or anemia. Research involving older adults and the relationship between vitamin D insufficiency and the development of anemia remains scarce. While this study found a positive association between these variables, further research is required before drawing causal conclusions, as the prevalence of anemia in older adults may be influenced by several factors, such as nutritional status and reduced sun exposure. The findings contribute to a more attentive and compassionate approach to older people, aiming to better guide health interventions for preventing and screening older adults at increased risk of anemia.

## 6. Conclusions

In this community-based sample of older adults, low serum vitamin D levels were associated with an increased risk of anemia. These findings are particularly relevant for public health, given the high prevalence of vitamin D insufficiency among Brazilian older adults. The results underscore the need to implement prevention and intervention strategies related to the co-occurrence of anemia and vitamin D insufficiency in older individuals, as these conditions can increase risks of morbidity and mortality and negatively impact quality of life. However, robust clinical trials are necessary to determine whether optimizing serum vitamin D levels can potentially reduce the prevalence of anemia in this population.

## Figures and Tables

**Table 1:** Percentage distribution of older adults according to socioeconomic aspects, health, vitamin D insufficiency, and anemia. Alfenas, 2019 (*n* = 430).

Variables	Total *n* (%)	Anemia		*p*
		No	Yes	
		*n* (%)	*n* (%)	
Sex				
Female	70.8	88.0	12.0	0.01
Male	29.2	78.6	21.4	
Age group				
60 to 69	45.3	88.2	11.8	0.24
70 a 79	38.4	83.7	16.3	
80 or older	16.3	80.8	19.2	
Years of education				
>4	33.9	82.1	17.9	0.25
≤4	66.1	86.5	13.5	
Marital status				
With partner	52.3	85.3	14.7	0.90
Without partner	47.7	84.9	15.1	
Living arrangement				
Does not live alone	81.5	85.1	14.9	0.79
Lives alone	18.5	83.9	16.1	
Household income				
>2 minimum wages ^a^	34.9	82.6	17.4	0.65
>1 e ≤ 2 minimum wages	44.3	86.3	13.7	
≤1 minimum wage	20.8	84.9	15.1	
Multimorbidity				
No	30.2	88.5	11.5	0.18
Yes	69.8	83.5	16.5	
Polipharmacy				
No	58.4	88.9	11.1	0.01
Yes	41.6	80.0	20.0	
Food insecurity screening				
Food-secure	86.4	86.3	13.7	0.10
Food-insecure	13.6	78.3	21.7	
Vitamin D insufficiency				
No	36.5	90.5	9.5	0.03
Yes	63.5	82.8	17.2	

^a^ In 2019, the minimum wage was BRL 998.00, approximately equivalent to USD 250.00.

**Table 2:** Univariate analysis of the association between vitamin D insufficiency and anemia. Alfenas, 2019 (*n* = 430).

Variables	OR ^b^	*p*	CI95% ^c^
Sex			
Female	1.0		
Male	2.0	0.01	1.16–3.41
Age group			
60 to 69	1.0		
70 a 79	1.4	0.21	0.80–2.61
80 or older	1.8	0.12	0.85–3.64
Years of education			
>4	1.0		
≤4	0.7	0.25	0.41–1.25
Marital status			
With partner	1.0		
Without partner	1.0	0.90	0.61–1.75
Living arrangement			
Does not live alone	1.0		
Lives alone	1.1	0.79	0.56–2.11
Household income			
>2 minimum wages ^a^	1.0		
>1 e ≤ 2 minimum wages	0.7	0.35	0.41–1.38
≤1 minimum wage	0.8	0.66	0.41–1.76
Food insecurity screening			
Food-secure	1.0		
Food-insecure	1.7	0.11	0.88–3.44
Multimorbidity			
No	1.0		
Yes	1.2	0.18	0.90–1.68
Polipharmacy			
No	1.0		
Yes	2.0	0.01	1.17–3.43
Vitamin D insufficiency			
No	1.0		
Yes	2.0	0.03	1.06–3.65

^a^ In 2019, the minimum wage was BRL 998.00, approximately equivalent to USD 250.00; ^b^ OR (Odds Ratio brute); ^c^ CI95% (confidence interval of 95%).

**Table 3:** Final multiple regression model of the association between vitamin D insufficiency and anemia. Alfenas, 2019 (*n* = 430).

Variables	OR ^a^	*p*	CI95% ^b^
Sex			
Female	1.0		
Male	2.7	0.01	1.5–4.9
Age group			
60 to 69	1.0		
70 a 79	1.7	0.10	0.9–3.2
80 or older	1.7	0.17	0.8–3.9
Polipharmacy			
No	1.0		
Yes	2.0	0.04	1.1–3.9
Food insecurity screening			
Food-secure	1.0		
Food-insecure	2.0	0.06	1.0–4.3
Multimorbidity			
No	1.0		
Yes	1.0	0.96	0.7–1.5
Vitamin D insufficiency			
No	1.0		
Yes	2.4	0.01	1.2–4.7

OR ^a^ (adjusted odds ratio); CI95% ^b^ (confidence interval of 95%).

## Data Availability

The original contributions presented in the study are included in the article; further inquiries can be directed to the corresponding authors.
